# Successful surgical management of intralobar pulmonary sequestration in Ghana

**DOI:** 10.1093/jscr/rjac085

**Published:** 2022-04-01

**Authors:** Isaac Okyere, Sandra Owusu Kwarteng, Atta Owusu Bempah, Perditer Okyere, Augustina Badu-Peprah, Samuel Gyasi Brenu

**Affiliations:** Department of Surgery, School of Medicine and Dentistry, College of Health Sciences, Kwame Nkrumah University of Science and Technology, Kumasi, Ghana; Department of Child Health, School of Medicine and Dentistry, College of Health Sciences, Kwame Nkrumah University of Science and Technology, Kumasi, Ghana; Directorate of Obstetrics and Gynecology, Komfo Anokye Teaching Hospital, Kumasi, Ghana; Department of Medicine, School of Medicine and Dentistry, College of Health Sciences, Kwame Nkrumah University of Science and Technology, Kumasi, Ghana; Department of Radiology, School of Medicine and Dentistry, College of Health Sciences, Kwame Nkrumah University of Science and Technology, Kumasi, Ghana; Directorate of Surgery, Komfo Anokye Teaching Hospital, Kumasi, Ghana

## Abstract

Pulmonary sequestration occurs when a portion of lung tissue receives its blood supply from an anomalous systemic artery. Three main presentations, intralobar, extralobar and communicating bronchopulmonary foregut malformations, have been described. It is the second most common congenital lung anomaly. The intralobar variant is the most common type seen in 75% of cases, especially in late childhood. Imaging of choice for diagnosis are computed tomography scan and magnetic resonance imaging. Management involves surgical resection with ligation of the aberrant blood supply via thoracotomy or thoracoscopy. Endovascular therapy with coil embolization of the aberrant anomalous systemic artery as a standard therapy or as a hybrid therapy is an option. We present our successful surgical management of an infant diagnosed prenatally with congenital lung abnormality and confirmed postnatally as intralobar pulmonary sequestration.

## INTRODUCTION

Pulmonary sequestration is a rare congenital lung malformation characterized by a non-communicating segment of lung tissue with an anomalous arterial blood supply [1]. It is the most common pulmonary malformation after congenital pulmonary adenomatoid malformation [2]. It typically presents with respiratory distress at birth [2, 3]. We present a case of an infant diagnosed prenatally with a lung mass suspected to be congenital pulmonary adenomatoid malformation but found postnatally as pulmonary sequestration both radiologically and pathologically.

## CASE SUMMARY

**Figure 1:**
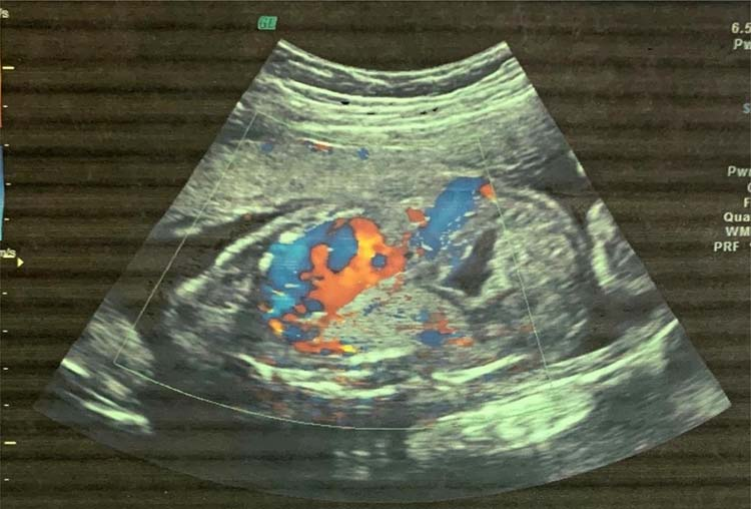
Fetal US at 20 weeks’ gestation showing an echogenic mass in the left lower lobe, which was initially thought to be congenital pulmonary adenomatoid malformation.

**Figure 2:**
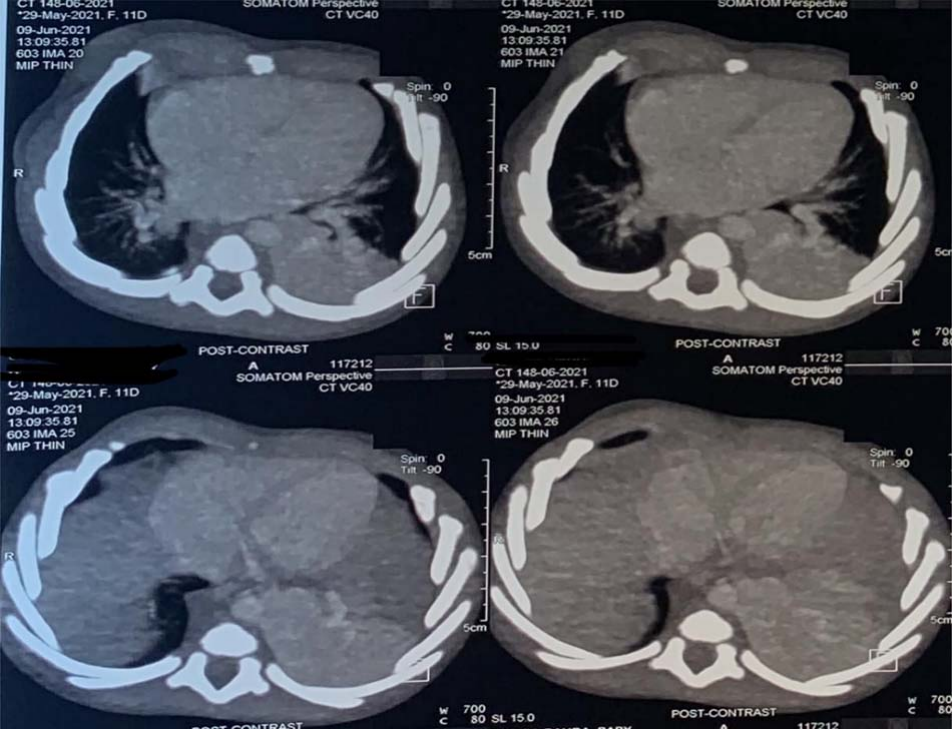
CT scan images depicting intralobar pulmonary sequestration of the left lower lung lobe.

A 6-month-old male baby who was found to have fetal adenomatoid malformation from a prenatal ultrasound (US) done at 20 weeks’ gestation and polyhydramnios at 30 weeks’ gestation (Fig. 1) is reported. The mass measured 4.7 × 4.1 × 3.5 cm with no evidence of systemic feeding artery, associated anomalies or hydrops at 24 weeks. Cesarean delivery was done at 38 weeks’ gestation for severe polyhydramnios. He spent first 3 weeks at the neonatology unit with respiratory distress and again on sixth week of life for pneumonia. A chest computed tomography (CT) scan (Fig. 2) at 2 weeks showed a wedge-shaped homogenously enhancing mass measuring 4.8 × 2.8 × 1.8 cm in the left lower lobe with an aberrant arterial branch from the descending thoracic at T12. Cardiothoracic surgery reviewed and maintained patient for surgery. He had normal preoperative laboratory work-up and underwent aberrant artery ligation and resection of the sequestrated lung via standard left posterolateral thoracotomy.

**Figure 3:**
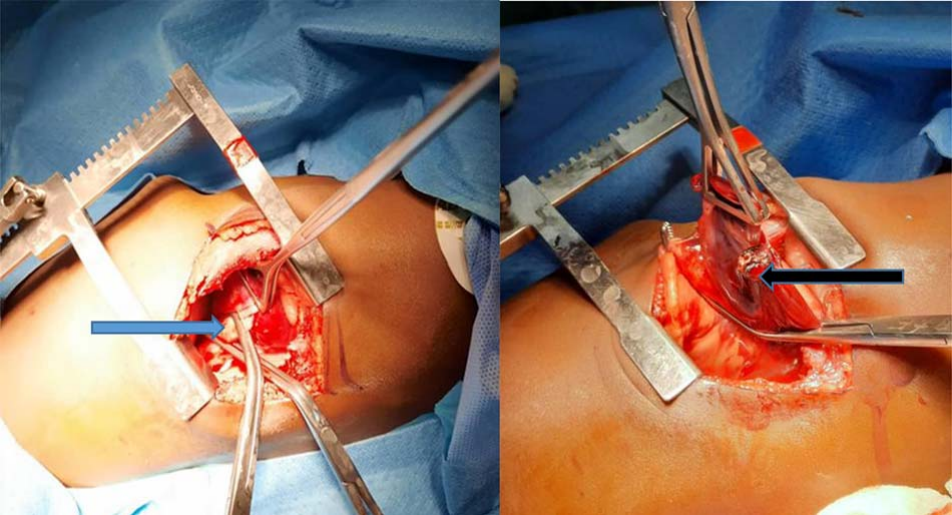
(**A**) Blue arrow showing the anomalous artery from the descending thoracic aorta; (**B**) black arrow showing the erythematous and dense sequestrated lung.

**Figure 4:**
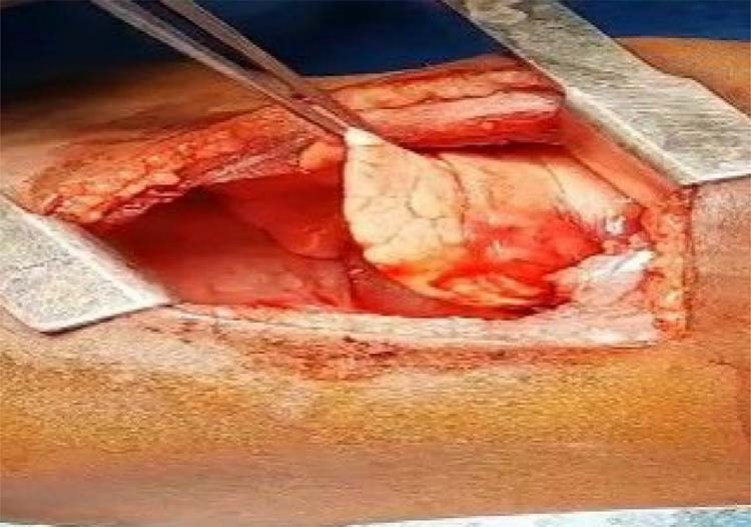
The normal lung after anomalous artery ligation and wedge resection of the sequestrated lung.

At surgery, the aberrant artery from the descending thoracic aorta (Fig. 3A) was dissected, isolated and doubly ligated with silk 1 and clips. A wedged resection of the sequestrated lung (Fig. 3B) was done, with Fig. 4 showing the normal lung after resection. No gastrointestinal tract communication was identified. A size 18F chest tube was left *in situ* after hemostasis. Patient was extubated on table and spent 2 days in the intensive care unit before being step down to the ward. The chest tube was removed on post-operative day 2 and he was discharged home on post-operative day 4 with subsequent follow-up on out-patient basis. He has been well with no complaints 6 months after surgery. Histopathologic evaluation of the resected sequestrated lung reported of thick-walled blood vessels in the lung parenchyma as well as evidence of hemorrhage and chronic inflammation within the alveolar spaces consistent with intralobar pulmonary sequestration.

## DISCUSSION

Pulmonary sequestration describes a congenital lung anomaly with an absent tracheobronchial tree and an anomalous systemic vasculature. Comprising 0.15–6.4% of all congenital lung malformations, pulmonary sequestration, though rare is the second most common congenital pulmonary anomaly with an incidence of 0.15–1.8% [2, 5]. There are mainly two types based on three characteristic features: the pleural investment, venous drainage and their location being intralobar and extralobar [6]. Intralobar sequestration is the most common type seen in 75% of cases [5]. They are prone to recurrent pneumonia [6]. Our patient with intralobar type was in the neonatal unit after birth for respiratory distress and again on sixth week of life for pneumonia. The intralobar pulmonary sequestration is usually located as noted in our case at the left posterior basal segment [5, 6] of the left lower lung lobe. An exception is where it is associated with Scimitar syndrome in which right-sided intralobar sequestrations have been described [7]. Extralobar sequestration, on the other hand, constitutes 25% of cases of pulmonary sequestration and is entirely separated from the normal lung parenchyma by its own pleural lining. Its venous drainage is via systemic veins such as azygos vein, hemiazygos vein, vena cava or even directly into the right atrium [2, 5, 6]. Systemic arterial supply is directly from the aorta, with the descending thoracic aorta being the most common with other rare origins like the abdominal aorta, celiac trunk, intercostal arteries, pericardiophrenic and coronary arteries [5, 7]. Our case had the anomalous artery (Fig. 3B) from the descending thoracic aorta. Intralobar sequestration contrary to the extralobar is usually acquired due to chronic inflammation of the pulmonary parenchyma with subsequent bronchial obstruction and acquisition of an aberrant systemic blood supply [2, 8]. Our rare case at 6 months of age lends more credence to the congenital etiology of intralobar sequestration similar to reports from Gabelloni and Laurin [2, 3]. A clinical suspicion of pulmonary sequestration should be made in a fetus found with polyhydramnios, hydrops fetalis or a pulmonary mass, or in an infant with recurrent episodes of pneumonia or in a child or young adult presenting with recurrent pneumonia, hemoptysis or lung abscess [7]. Prenatal diagnosis is possible as early as 16–18 weeks with confirmation of systemic arterial supply via fetal doppler US [1, 7]. Our patient had an echogenic mass identified at 20 weeks’ gestation, which was postnatally better visualized by contrast-enhanced CT scan. Magnetic resonance angiography is preferred in the pediatric population over CT scan since ionizing radiation is not utilized.

Mainstay of treatment involves resection with ligation of the aberrant arterial supply [1, 2] and isolation and ligation of any associated foregut communication [1]. Surgical resection by lobectomy or wedge resection as done in our case is required to halt the risk of recurrent infection, potential hemorrhage and malignant transformation [1, 2, 5].

Endovascular therapy with occlusion agents, such as polyvinyl alcohol, gelatin foams, Amplatzer occlusion devices and coils, are alternative options [1, 2, 9]. It is also valuable as a preoperative procedure to minimize intraoperative bleeding [2]. It has a high recurrence rate ranging from ~25 to 47% possibly due to incomplete occlusion or development of collaterals and therefore should be combined with open resection [2, 9].

Prenatal intervention, including thoracocentesis or thoracoamniotic shunting and antenatal steroids, US-guided intra-fetal radiofrequency ablation and laser coagulation, can also be employed, especially in those with hydrothorax or fetalis [4, 6]. Antenatal transplacental steroids were administered at 32 weeks’ gestation for our patient with no effect.

## CONCLUSION

Though intralobar pulmonary sequestration is a rare congenital lung anomaly, successful surgical management even in resource-restraint centers is possible. Treatment modalities include open surgery via thoracotomy, endovascular coil embolization or combination therapy.
